# Prevalence of Serologic Weak D Phenotype in Iranian Blood Donors: A Nationwide Retrospective Cross‐Sectional Study

**DOI:** 10.1002/hsr2.73271

**Published:** 2026-09-23

**Authors:** Younes Sadeghi‐Bojd, Narges Sadat Razavi Alhosayni, Fatemeh Mirzaeyan, Fatemeh Mohammadali, Arezoo Oodi

**Affiliations:** ^1^ High Institute for Research and Education in Transfusion Medicine Iranian Blood Transfusion Organization Tehran Iran

**Keywords:** blood donors, prevalence, RhD typing, serologic, Weak D phenotype

## Abstract

**Background and Aim:**

The serologic prevalence of the Weak D phenotype varies across populations and may be influenced by laboratory methodology. However, nationwide data from Iran remain limited. This study aimed to determine the nationwide serologic prevalence of the Weak D phenotype among Iranian blood donors and to compare detection rates between manual and automated RhD typing methods.

**Methods:**

This nationwide retrospective cross‐sectional study included 1,991,758 blood donors from all 31 provinces of Iran between December 2022 and December 2023. RhD typing was performed using either manual tube testing or automated platforms (Magnet and solid‐phase red cell adherence [SPRCA]). Prevalence estimates with 95% confidence intervals (CIs) were calculated and compared using *χ* ^2^ and paired analyses.

**Results:**

Among 1,991,758 blood donors, 213,693 (10.72%) were Rh‐negative. Serologic Weak D was identified in 1001 donors, corresponding to an overall prevalence of 0.0503% (95% CI: 0.0472–0.0535) and 0.4684% among Rh‐negative donors. The prevalence was 0.0491% with manual testing and 0.0518% with automated testing, with no significant difference between the two methods (*χ* ^2^ = 0.681, *p* = 0.41). Within the automated group, SPRCA demonstrated a significantly higher detection rate than the Magnet platform (0.0619% [95% CI: 0.0545–0.0707] vs. 0.0445% [95% CI: 0.0390–0.0507]; *χ* ^2^ = 12.227, *p* < 0.001). In provinces where both methods were used concurrently, paired analysis showed a significantly higher mean detection rate with automated testing (*p* = 0.023).

**Conclusion:**

The nationwide prevalence of serologically detected Weak D phenotype in Iranian blood donors was 0.0503%, placing Iran within the lower range of global estimates. Although no significant overall difference was observed between manual and automated RhD typing, the Solid Phase platform demonstrated significantly higher detection rates than the Magnet platform. These findings support standardized RhD typing strategies to improve diagnostic consistency and optimize transfusion safety at the national level.

## 1 Introduction

The Rh blood group system is one of the most clinically significant polymorphic antigen systems in human populations, second only to the ABO system in transfusion medicine and the prevention of hemolytic disease of the fetus and newborn (HDFN) [1]. Among the Rh antigens, the D antigen is highly immunogenic and plays a central role in alloimmunization risk. Accurate determination of RhD status is therefore critical for safe transfusion practice and obstetric management [1]. The RHD gene exhibits considerable genetic diversity, including deletions, hybrid RHD‐CE constructs, and point mutations that can reduce or modify D antigen expression [1, 2, 3]. These genetic variations give rise to distinct phenotypes, including weak D and partial D, which have important implications for clinical management [1, 3].

The weak D phenotype is characterized serologically by diminished reactivity with standard anti‐D reagents, while maintaining overall D antigenicity. Molecularly, Weak D usually results from single nucleotide variants in the RHD gene that reduce antigen density on erythrocytes without eliminating epitopes. Clinically, Weak D is significant because misclassification as Rh‐negative may lead to unnecessary administration of Rh immunoglobulin in pregnant women or inappropriate transfusion of Rh‐negative blood, whereas misclassification as Rh‐positive could theoretically increase alloimmunization risk in Rh‐negative recipients. Consequently, understanding the prevalence and distribution of Weak D is essential for optimizing transfusion strategies and immune prophylaxis policies [1, 3, 4, 5, 6].

Reported serologic prevalence of Weak D phenotypes differs among geographic regions and study populations [3, 7]. In European Caucasian populations, the majority of Weak D phenotypes are types 1, 2, and 3, with reported serologic frequencies ranging from 0.2% to 0.5% [3, 8]. In North America, similar prevalence has been documented; however, the adoption of molecular RHD genotyping has refined RhD classification, enabling more precise distinction between weak and partial D variants [3, 9]. Asian populations generally exhibit lower but heterogeneous frequencies, reflecting different molecular backgrounds and the diversity of RHD alleles in these populations [7, 10]. In the Middle East, reported serologic prevalence ranges from 0.03% to 0.2%, although comprehensive nationwide data are limited [11, 12, 13, 14, 15, 16, 17].

Accurate detection of Weak D remains method‐dependent. Traditional serologic approaches, such as manual tube indirect antiglobulin testing, have served as reference methods but are limited by operator variability and subjective interpretation [18, 19]. Automated systems, including gel card, solid‐phase red cell adherence (SPRCA), and magnet‐based platforms, offer increased standardization, improved reproducibility, and enhanced sensitivity [16, 20, 21]. Among these, SPRCA has shown high analytical sensitivity for detecting low‐density antigens due to controlled incubation conditions, standardized optical interpretation, and enhanced antigen–antibody interactions [20, 21, 22]. Variability in reagent composition, incubation conditions, and detection algorithms can significantly affect Weak D identification and reported prevalence, underscoring the importance of standardized testing [22].

Given the epidemiologic importance of the Weak D phenotype and the methodological variability in its detection, establishing robust population‐level prevalence data is essential for accurate interregional comparison and methodological consistency. Although prevalence studies have been reported in selected populations worldwide, nationwide serologic prevalence data in Iran remain limited. Therefore, this study aimed to determine the nationwide serologic prevalence of the Weak D phenotype in a large Iranian blood donor population and to evaluate differences in detection rates according to testing methodology.

## 2 Material and Methods

### 2.1 Study Design and Population

This nationwide retrospective cross‐sectional study utilized validated blood donor records from all 31 provincial blood transfusion centers across Iran between December 2022 and December 2023. The study was registry‐based and included all eligible donor records during the study period. To ensure accurate prevalence estimation and avoid duplicate counting, each donor was included only once. For donors with multiple donations during the study period, only the first validated donation record was retained. Donor identification numbers were used to identify repeat donations. In accordance with the Iranian Blood Transfusion Organization donor testing policy, any detectable weak expression of the D antigen was classified as Weak D in blood donors. Therefore, repeated donations did not influence the estimated prevalence of the Weak D phenotype. Both first‐time and regular donors were included, while records with missing, inconsistent, or duplicate information were excluded.

### 2.2 Serologic Testing

Serologic Testing RhD typing was performed either manually or using automated platforms according to the standard protocols of the Iranian Blood Transfusion Organization. Manual RhD typing was initially performed using monoclonal Anti‐D reagent (IgM/IgG; clones RUM‐1 and MS‐26; Lorne Laboratories, UK). Samples showing no agglutination or an agglutination reaction of < 2+ at the immediate‐spin (IS) phase were subsequently re‐evaluated by the conventional tube method using Blend Anti‐D (human IgM/IgG; clones TH‐28 and MS‐26; Immucor Diagnostik GmbH, Germany) and were further tested by the indirect antiglobulin test (IAT) using anti‐human globulin (AHG) reagent (Immucor Diagnostik GmbH, Germany). Automated RhD typing was performed using the Qwalys (Diagast, France) and NEO Iris (Immucor, USA) systems with the anti‐D reagents supplied by the respective manufacturers according to the manufacturers' instructions. Samples demonstrating weak or inconclusive RhD reactivity and confirmed by the IAT/AHG procedure were classified as serologic Weak D phenotypes. As this nationwide study relied exclusively on serologic testing, molecular RHD genotyping was not performed; therefore, serologic methods could not distinguish weak D from partial D or other RHD variants. Consequently, all prevalence estimates reported in this study represent serologically detected Weak D phenotypes.

### 2.3 Statistical Analysis

Prevalence rates were calculated and expressed as percentages. Ninety‐five percent confidence intervals (95% CIs) for prevalence estimates were calculated using the Wilson score method. Categorical variables were compared using the *χ* ^2^ test. The normality of continuous variables was assessed using the Shapiro–Wilk test. In provinces where both manual and automated methods were employed, paired *t*‐tests were used to compare Weak D detection rates. All statistical tests were two‐sided, and a *p* value < 0.05 was considered statistically significant. Statistical analyses were performed using IBM SPSS Statistics for Windows, Version 26.0 (IBM Corp., Armonk, NY, USA). Statistical analyses and reporting were performed in accordance with the SAMPL (Statistical Analyses and Methods in the Published Literature) guidelines. Standard statistical terminology, abbreviations, and symbols were used throughout the manuscript. All statistical analyses were prespecified according to the study objectives, and no post hoc exploratory or subgroup analyses were performed.

### 2.4 Ethical Considerations

The study protocol was approved by the Medical Ethics Committee of the High Institute for Research and Education in Transfusion Medicine, Tehran, Iran (IR.TMI.REC.1404.013). All donor data were anonymized prior to analysis to ensure confidentiality. The study was conducted in accordance with the principles of the Declaration of Helsinki and relevant national regulations for research involving human participants.

## 3 Results

### 3.1 Study Population

A total of 1,991,758 blood donors from 31 provinces of Iran were included in the analysis. Among them, 213,693 (10.72%) were Rh‐negative. Overall, 1,001 donors were identified with the serologic Weak D phenotype, corresponding to an overall prevalence of 0.0503% (95% CI: 0.0472% to 0.0535%). Among Rh‐negative donors, the prevalence of the serologic Weak D was 0.4684% (Table 1).

**Table 1:** Distribution of the serologic Weak D phenotype according to RhD typing method.

Methods	Donors (*n*)	Rh‐Negative (*n*)	WeakD (*n*)	Rh‐Negative (%)	WeakD (%)	WeakD/Rh‐Negative (%)	*χ* ^2^	*p* value
Manual	1,137,977	121,878	559	10.71	0.049	0.45	0.681	0.41
Automated	853,781	91,815	442	10.75	0.051	0.48		
Total	1,991,758	213,693	1,001	10.72	0.050	0.46		

### 3.2 Comparison Between Manual and Automated Methods

Of the total donors, 1,137,977 were tested using the manual method and 853,781 using automated systems. The prevalence of the serologic Weak D was 0.0491% (95% CI: 0.04399% to 0.04989%). In the manual method and 0.0518% in the automated method. *χ* ^2^ analysis demonstrated no statistically significant difference between the two approaches (*χ* ^2^ = 0.681, *p* = 0.41) (Table 1).

### 3.3 Comparison Between Two Automation Techniques

Within the automated testing group, 496,913 samples were screened using the Magnet method and 356,868 using the Solid Phase Red Cell Adherence (SPRCA) method. The prevalence of serologic Weak D was 0.0445% (95% CI: 0.0390–0.0507) with the Magnet method and 0.0619% (95% CI: 0.0545–0.0707) with the SPRCA method. This difference was statistically significant (*χ* ^2^ = 12.227, *p* < 0.001), indicating a higher detection rate with the SPRCA method (Table 2). Although both the Magnet and SPRCA platforms identified the same absolute number of serologic Weak D cases (*n* = 221), the total numbers of donors tested by each platform differed substantially (496,913 and 356,868, respectively). Therefore, the observed difference in prevalence reflects the unequal denominators rather than any inconsistency in the study data.

**Table 2:** Comparison between Magnet and Solid Phase automation systems.

Automation technique	Donors (*n*)	Rh‐Negative (*n*)	WeakD (*n*)	Rh‐Negative (%)	WeakD (%)	WeakD/Rh‐Negative (%)	*χ* ^2^	*p* value
Magnet	496,913	52,323	221	10.5	0.04	0.42	12.227	< 0.001
SPRCA^a^	356,868	39,492	221	11.06	0.06	0.55		
Total	853,781	91,815	442	10.75	0.051	0.48		

^a^ Solid‐Phase Red Cell Adherence.

### 3.4 Paired Analysis in Provinces Using Both Methods

Eighteen provinces used both manual and automated methods concurrently. Normality testing of paired differences using the Shapiro–Wilk test showed normal distribution (*W* = 0.955, Degree of Freedom (DF) = 18, *p* =;0.52). Paired *T*‐test analysis demonstrated a statistically significant difference between methods (*t* = −2.493, DF = 17, *p* = 0.023). The mean prevalence was 0.0457% for the manual method and 0.0717% for the automated method. The mean difference was −0.0260% (95% CI: −0.0480 to −0.0040). A significant positive correlation between the two methods was also observed (*r* = 0.688, *p* = 0.002) (Table 3). The prevalence estimate obtained from the paired provincial analysis (0.0717%) should not be directly compared with the overall nationwide automated prevalence (0.0518%), because the two estimates were derived from different analytical populations. The nationwide automated prevalence was calculated using all donors tested by automated platforms across the 31 participating provinces, whereas the paired analysis was restricted to the 18 provinces in which both manual and automated RhD typing methods were performed concurrently. This paired design minimized geographic confounding and provided a more appropriate methodological comparison between manual and automated RhD typing.

**Table 3:** Paired *T*‐test comparing manual and automated methods.

Statistic	Value
Mean manual prevalence (%)	0.0457
Mean automated prevalence (%)	0.0717
Mean difference (%)	−0.0260
SD^a^ of difference	0.0442
*T*	−2.493
DF^b^	17
*p*‐value	0.023
95% CI	−0.0480 to −0.0040
Correlation (*r*)	0.688
Correlation *p*‐value	0.002

^a^ Standard Deviation.

^b^ Degree of Freedom.

### 3.5 Comparison of Nationwide and Paired Provincial Analyses

These findings indicate that the apparent discrepancy between the nationwide analysis (Table 1) and the paired provincial analysis (Table 3) is attributable to differences in study design rather than inconsistent results. While the nationwide analysis reflects overall serologic Weak D prevalence across all participating provinces, the paired analysis isolates the effect of testing methodology by comparing manual and automated RhD typing within the same provinces. Therefore, the paired provincial analysis provides the most appropriate estimate for evaluating the relative performance of the two testing methods.

## 4 Discussion

This nationwide retrospective study provides the first large‐scale, population‐based estimate of serologic Weak D prevalence among blood donors in Iran. Using validated and deduplicated data collected over a 1‐year period, the overall prevalence of the serologic Weak D was 0.0503%, while 0.4684% of Rh‐negative donors demonstrated Weak D reactivity. To date, no nationwide serologic prevalence study has been reported from Iran; previously published Iranian investigations have primarily focused on molecular characterization of RHD variants rather than epidemiologic prevalence assessment [1, 2, 4]. Our findings therefore establish an essential national baseline for future comparative and molecular studies.

Globally, the prevalence of serologic Weak D ranges from 0.02% to 1%, depending on ethnicity, population genetics, and testing methodology [3, 7]. In European populations, prevalence among blood donors is typically reported between 0.2% and 0.5%, reflecting the predominance of Weak D phenotypes are types 1, 2, and 3, in Caucasian cohorts [3, 8]. In North America, similar prevalence rates have been described; however, implementation of RHD genotyping has refined RhD classification and improved transfusion management strategies [3, 9]. In contrast, Asian populations generally demonstrate lower but heterogeneous prevalence rates. Chinese studies report frequencies between 0.02% and 0.1%, with distinct molecular backgrounds compared with European populations [23, 24]. Indian data indicate prevalence between 0.09% and 0.3%, with marked regional variation [17, 25]. Reports from Middle Eastern countries remain limited but typically fall within 0.03% to 0.2%, influenced by ethnic diversity and laboratory methodology [11, 12, 13, 14, 15, 16]. The nationwide prevalence observed in the present study (0.0503%) places Iran within the lower spectrum of global estimates and closer to East Asian and Gulf populations than to European cohorts.

Significant geographic heterogeneity was observed across Iranian provinces, with prevalence ranging from 0.0029% to 0.1498%. Such variability likely reflects underlying ethnic diversity and differential distribution of RHD alleles. Similar intra‐country variation has been reported in multi‐ethnic populations and attributed to ancestral genetic structure and founder effects. Given Iran's ethnically diverse demographic composition, regional variability in RHD variant distribution is biologically plausible and warrants molecular epidemiologic investigation [1, 2, 4].

Accurate detection of Weak D remains method‐dependent. Traditional serologic approaches, such as manual tube indirect antiglobulin testing, have served as reference methods but are limited by operator variability and subjective interpretation [18, 19]. Automated systems, including gel card, SPRCA, and magnet‐based platforms, improve standardization, reproducibility, and analytical consistency in RhD typing [16, 20, 21]. Among these, solid phase technologies have been associated with greater analytical sensitivity for detecting weak antigen expression [20, 21, 22]. Variability in reagent formulation, incubation parameters, and detection algorithms may substantially influence serologic Weak D identification and consequently affect reported prevalence rates [22].

Beyond epidemiologic distribution, the methodological dimension of Weak D detection warrants careful consideration. Serologic identification is inherently influenced by reagent sensitivity and platform characteristics [3]. Although the manual tube indirect antiglobulin test has traditionally served as the reference approach, automation has progressively replaced manual techniques to enhance consistency and workflow efficiency [18, 19]. Comparative evaluations demonstrate strong concordance between solid phase method and manual indirect antiglobulin testing in Weak D detection [20, 21, 22], while automated gel technologies reduce subjectivity in grading and improve reaction stability [26, 27].

Direct comparative evidence between Magnet‐based method and solid phase platforms in Weak D detection remains limited. However, broader evaluations of automated immunohematology analyzers suggest that detection yield may vary according to analytical sensitivity [20, 21, 22]. In our nationwide dataset, solid phase automation identified significantly more Weak D cases than Magnet method (0.0619% vs. 0.0445%, *p* < 0.001), supporting the interpretation that methodological differences contribute to variation in observed prevalence. However, because the distribution of testing platforms differed across provinces, crude nationwide comparisons may partly reflect regional differences in genetic background and the distribution of RHD variants rather than analytical performance alone. Therefore, the paired provincial analysis, restricted to provinces where both manual and automated methods were used concurrently, provides a more appropriate comparison by minimizing geographic confounding. Nevertheless, some residual confounding may still exist. Future studies based on individual‐level data should consider mixed‐effects regression models with province included as a random effect to further account for regional heterogeneity.

In summary, this study provides the first nationwide estimate of the prevalence of serologically detected Weak D among Iranian blood donors. Although no significant difference was observed between manual and automated RhD typing overall, the significantly higher detection rate achieved with the Solid Phase platform compared with the Magnet platform indicates that analytical platform selection may influence the reported prevalence of serologic Weak D. These findings support the implementation of standardized RhD typing strategies and unified national testing algorithms to improve diagnostic consistency, optimize Rh‐negative blood utilization, and strengthen transfusion safety in Iran.

### 4.1 Limitations

This study has several limitations. First, the study was based on routinely collected nationwide registry data, and the distribution of testing platforms differed across provinces. Although a paired provincial analysis was performed to minimize geographic confounding, some residual confounding related to regional genetic background and the distribution of *RHD* variants may still have influenced the nationwide comparisons. Second, the study relied exclusively on serologic testing, and molecular *RHD* genotyping was not available. Consequently, serologically detected Weak D phenotypes could not be differentiated from partial D, DEL, or other *RHD* variants with different clinical significance. Third, because the analyses were based on aggregated registry data rather than individual‐level observations, more advanced statistical approaches, such as mixed‐effects regression models accounting for province‐level variation, could not be applied. Fourth, longitudinal information on individual donors across multiple donation visits was not available; therefore, we were unable to assess whether RhD classifications changed or differed between separate donation visits. Future nationwide studies integrating molecular characterization and individual‐level longitudinal analyses would provide a more comprehensive understanding of the epidemiology and molecular spectrum of Weak D phenotypes in the Iranian donor population.

## 5 Conclusion

This first nationwide assessment of serologic Weak D prevalence among Iranian blood donors demonstrates an overall prevalence of 0.0503%, with 0.4684% among Rh‐negative individuals. Considerable regional variation was observed across provinces. Although no overall difference was found between manual and automated testing, solid‐phase automation showed significantly higher detection rates compared with Magnet‐based method, highlighting the influence of analytical platform sensitivity on reported prevalence. These findings establish a national epidemiologic baseline and underscore the importance of standardized, high‐sensitivity automated RhD typing strategies to enhance diagnostic accuracy, optimize Rh‐negative blood utilization, and strengthen transfusion safety policies in Iran.

## Author Contributions

**Younes Sadeghi‐Bojd:** conceptualization, validation, writing – review and editing. **Narges sadat Razavi alhosayni:** conceptualization and investigation. **Fatemeh Mirzaeyan:** project administration. **Fatemeh Mohammadali:** project administration. **Arezoo Oodi:** conceptualization, investigation, writing – review and editing, validation, methodology, project administration, supervision, data curation, formal analysis, funding acquisition.

## Disclosure

All authors have read and approved the final version of the manuscript. Arezoo Oodi, the corresponding author, had full access to all of the data in this study and takes complete responsibility for the integrity of the data and the accuracy of the data analysis.

## Conflicts of Interest

The authors declare no conflicts of interest.

## Transparency Statement

The lead author, Arezoo Oodi, affirms that this manuscript is an honest, accurate, and transparent account of the study being reported; that no important aspects of the study have been omitted; and that any discrepancies from the study as planned (and, if relevant, registered) have been explained. Arezoo Oodi affirms that this manuscript is an honest, accurate, and transparent account of the study being reported; that no important aspects of the study have been omitted; and that any discrepancies from the study as planned have been explained.

## Data Availability

The data that support the findings of this study are available from the corresponding author upon reasonable request. The raw data supporting the conclusions of this article will be made available by the authors on request.
